# Cervical Actinomycosis Diagnosed via Metagenomic Next-Generation Sequencing of Formalin-Fixed Paraffin-Embedded Tissue: A Case Report and Literature Review

**DOI:** 10.3390/microorganisms13081855

**Published:** 2025-08-08

**Authors:** Teresa K. F. Wang, Hin-Fung Tsang, Sze Chuen Cesar Wong, Stanley W. M. Leung

**Affiliations:** 1 Hong Kong Medical Consultants Limited, Hong Kong SAR, China; 2 Department of Clinical Laboratory and Pathology, Hong Kong Adventist Hospital, Hong Kong SAR, China; 3 Department of Applied Biology & Chemical Technology, The Hong Kong Polytechnic University, Hong Kong SAR, China

**Keywords:** cervical actinomycosis, *Actinomyces*, metagenomic, next-generation sequencing, formalin-fixed paraffin-embedded tissue, head and neck

## Abstract

Actinomycosis is an uncommon but significant chronic bacterial infection affecting various parts of the body caused by *Actinomyces* species. Because of the nonspecific symptoms and rarity of the condition, the diagnosis of head-and-neck or cervicofacial actinomycosis is usually challenging and delayed. A 39-year-old woman presented with an enlarging right neck mass and dysphagia after steroid exposure for treatment of De Quervain thyroiditis. MRI showed a large irregular infiltration mass over the right side of her neck, with a multi-loculated rim-enhancing area over the right retropharyngeal space. Excisional biopsy of the lesion only showed evidence of acute on chronic inflammation, and the results of all microbiological testing (including bacterial culture, Gram-staining, and molecular detection) were negative. Metagenomic next-generation sequencing (mNGS) of the formalin-fixed paraffin-embedded (FFPE) tissue from the patient was performed. DNA of *Actinomyces israelii* and *Methylobacterium* was detected. The patient was confirmed to have cervical actinomycosis and completely recovered after 6 months of oral amoxicillin. Our patient is the first case utilizing mNGS on FFPE tissue to diagnose cervical actinomycosis. This case shows that mNGS is a promising, unbiased tool for detecting *Actinomyces* species in FFPE tissues and diagnosing cervical actinomycosis. It also highlights the diagnostic difficulties of cervical actinomycosis.

## 1. Introduction

*Actinomyces* species are Gram-positive, non-spore-forming, non-motile, anaerobic to microaerophilic filamentous bacilli. They can be found in the oral cavity, genitourinary tract, and gastrointestinal tract [1,2]. *Actinomyces* species are a predominant bacterial species in the oral cavity, where they are considered normal oral microbiota [3]. In certain situations that compromise the anatomical barrier and increase host susceptibility, *Actinomyces* species may nevertheless become pathogenic. It leads to a condition known as actinomycosis, an uncommon but significant chronic bacterial infection primarily caused by *Actinomyces* species, most commonly *Actinomyces israelii* [1]. This infection can affect various parts of the body, including the cervicofacial, abdominopelvic, and thoracopulmonary regions [2]. The clinical presentation of actinomycosis is often nonspecific and may mimic several diseases such as malignancy, tuberculosis, nocardiosis, and other chronic inflammatory diseases [3,4]. These presentations include the formation of indurated masses, sometimes with draining sinuses that may produce thick and yellowish discharge (with sulfur granules); the formation of painful swelling and abscess; or the presence of fever or malaise. Cervicofacial actinomycosis is generally considered an endogenous infection because the causative agents, *Actinomyces* species, are commensals of the human oropharynx [3]. In most cases, cervicofacial actinomycosis is caused by mucosal lesions [5] and involves the tissues surrounding the upper or lower mandible, causing large abscesses. In some cases, bone and mandibular joint involvement are observed, resulting in mandibular osteomyelitis [3].

The diagnosis of actinomycosis is challenging due to its nonspecific symptoms and rarity [3]. As a result, the diagnosis of head-and-neck or cervicofacial actinomycosis is usually delayed. Diagnoses can be made during clinical examinations when there is a compatible history and discharging sinuses upon physical examination. Imaging studies, such as computed tomography (CT) or magnetic resonance imaging (MRI), are useful to assess the location and extent of infection, and microbiological cultures from biopsies or aspirates help to confirm the presence of *Actinomyces* species [3]. However, the failure rate of bacterial culture is high [6,7,8] due to inhibition of bacterial growth by previous antibiotic therapy, contamination by other microorganisms, or the use of inappropriate culture media or culture conditions [3]. Chocolate blood agar or other enriched media, such as brain heart infusion broth and Brucella blood agar with hemin and vitamin K1, should be used as culture media [3]. The growth rate of *Actinomyces* species is slow, requiring incubation for at least 10 days under an anaerobic atmosphere before confirming the absence of *Actinomyces* [3]. Most cases in the literature are diagnosed solely based on clinical and histopathological findings, without any microbiological assessment.

The introduction of molecular detection to facilitate early diagnosis of this slowly progressing actinomycosis can help clinicians to administer timely and appropriate treatments, avoiding morbidity and unwanted surgery. In recent years, metagenomic next-generation sequencing (mNGS) has revolutionized the field of clinical microbiology by allowing a direct, simultaneous, and unbiased detection of all pathogens, including bacteria, fungi, viruses, and parasites, in a single clinical specimen [9,10]. While targeted molecular diagnostic approaches using polymerase chain reaction (PCR) rely on prior suspicion and knowledge of pathogens’ genomes, mNGS does not have limitations regarding the detection of specific pathogens. It allows an untargeted and hypothesis-free detection of any pathogens in a clinical specimen [10]. The advent of sequencing-based diagnostic tools has opened up new avenues to overcome culture delays in the diagnosis of infectious diseases, especially for slow-growing organisms. It is best illustrated in previous studies of mNGS associated with *Mycobacterium tuberculosis*. In a study that compared the diagnostic performances of mNGS, PCR, *Mycobacterium tuberculosis* (MTB) culture, and Ziehl–Neelsen (ZN) staining using cerebrospinal fluid (CSF) for tuberculous meningitis, Chen et al. showed that mNGS has the highest sensitivity among all diagnostic methods [11]. However, mNGS was not recommended for use as a rule-out test [11]. In another comparative study, Yu et al. compared the diagnostic accuracy of nanopore sequencing, PCR, MTB culture, and ZN staining for the diagnosis of pulmonary tuberculosis (PTB) using respiratory samples [12]. The diagnostic accuracy of nanopore sequencing for PTB was excellent, with the highest sensitivity among the four methods and comparable specificity to PCR and bacterial culture [12]. These studies showcased the high value and excellent performance of NGS-based methods in the diagnosis of infectious diseases. In this case report, we present a case of cervical actinomycosis diagnosed via mNGS on formalin-fixed paraffin-embedded (FFPE) tissue from the patient.

## 2. Case Presentation

A 39-year-old woman presented with an enlarging right neck mass with dysphagia in April 2024. She had a history of biopsy-confirmed De Quervain thyroiditis, diagnosed in February 2024, and was given prednisolone 30 mg daily starting on 27 February 2024 for 4 weeks, followed by prednisolone 20 mg daily for 1 week, before she noticed the presence of a mass on the right side of her neck. The dosage of prednisolone was increased to 30 mg daily again for 10 days and then was tapered down to 15 mg daily on 13 May 2024; all steroids were stopped on 21 May 2024. The right neck mass showed no reduction in size.

**Figure 1:**
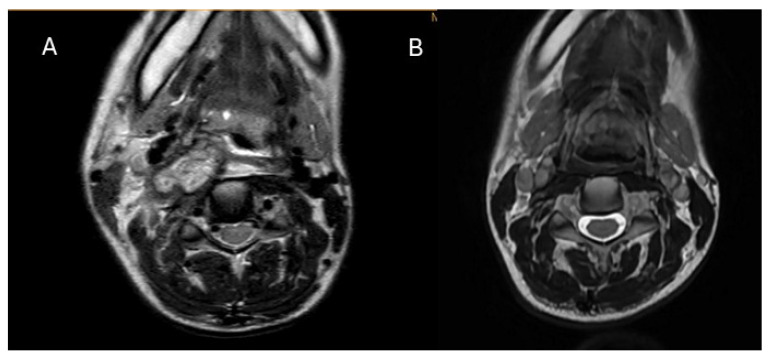
(**A**) T2-weighted axial MRI at the level of the mandible. It shows the presence of an irregular infiltration mass over the right side with extension to the retropharyngeal space and carotid space before treatment started. (**B**) MRI was repeated, and the mass resolved 3 months after starting appropriate treatment.

On 6 June 2024, her total white blood cell count was 9.5 × 10^9^/L (normal interval: 3.7–9.3 × 10^9^/L), with 7.5 × 10^9^/L neutrophils and 2.0 × 10^9^/L lymphocytes. Her liver, renal, and thyroid function were all normal, but her erythrocyte sediment rate (ESR) and C-reactive protein (CRP) level were elevated to 112 mm/h (normal interval: 0–31 mm/h) and 12.2 mg/L (normal interval: 0–4.9 mg/L), respectively. MRI was performed on her neck, showing a large irregular infiltration mass over the right side of her neck, with a multi-loculated rim-enhancing area over the right retropharyngeal space, 4.1 × 1.9 × 4.8 cm in size (Figure 1A). The mass extended from the right retropharyngeal space just posterior to the right tonsil to the right carotid space, encasing the right common carotid artery and the internal jugular vein, and further anteriorly to the subcutaneous region of the right anterior neck, with infiltration into the right sternocleidomastoid muscle, at a size of 3.3 × 2.3 × 3.0 cm. A Tru-cut biopsy was performed on 6 June 2024, showing granulation tissue and mixed inflammatory infiltrates with occasional multinucleated giant cells without granulomatous inflammation. The bacterial culture and PCR for *Mycobacterium tuberculosis* complex were negative. Blood for the interferon-gamma releasing assay was negative. She was given moxifloxacin 400 mg daily for one week but showed no improvement. Blood tests were repeated on 17 June 2024, showing further elevation of ESR to ≥120 mm/h, CRP of 20.1 mg/L, and neutrophil counts of 9.5 × 10^9^/L. In view of the negative microbiological investigation results and the lack of response to antibiotics, an excisional biopsy was performed on 26 June 2024. The histology findings showed acute on chronic inflammation of the skin, with granulation tissue proliferation and fibrosis of the underlying soft tissue. Gram-staining showed a small number of Gram-positive cocci in clusters, but no specimen was sent for culture. Azithromycin was started on 24 June 2024 until 2 July 2024. Wound healing was delayed over the excision site, with persistent whitish chalk-like discharge. The antibiotic was switched to linezolid 600 mg twice daily from 3 to 22 July. A wound swab sample was taken on 5 July 2024, which showed no bacterial growth after 5 days of incubation at 35 °C under both aerobic and anaerobic conditions.

In view of the Gram-stain findings from the tissue taken on 26 June 2024 and the lack of bacterial culture for correlation, a FFPE specimen was retrieved, and mNGS was performed to detect any potential pathogens. In the mNGS analysis, 15 FFPE slides, each 5 µm thick, were prepared from the tissue block specimen. The tissue was deparaffinized with mineral oil for molecular biology (cat. M5904-500 mL) (Sigma-Aldrich, Darmstadt, Germany) and incubated at 80 °C for 3 min. DNA was extracted from the FFPE tissue using a Qiagen Allprep DNA/RNA FFPE kit (cat. 80234) (Qiagen, Hilden, Germany), according to the manufacturer’s instructions. The concentration of the extracted DNA was evaluated using a Qubit 1X dsDNA HS Assay Kit (cat. Q33230) (Invitrogen, Waltham, MA, USA), and the quality was evaluated using an Agilent genomic DNA ScreenTape system (cat. 5067-5365) (Agilent Technologies, Barcelona, Spain). DNA libraries were prepared using the KAPA HyperCap FFPET DNA protocol (cat. KK8510) (Roche, Basel, Switzerland) for metagenomics sequencing according to the manufacturer’s instructions, except that a universal adaptor was used instead of a UMI adaptor. The prepared libraries were quantified using a Qubit 1X dsDNA HS Assay Kit (cat. Q33230) (Invitrogen, Waltham, MA, USA) and were qualified using an Agilent D1000 ScreenTape system (cat. 5067-5582) (Agilent Technologies, Barcelona, Spain). The prepared DNA libraries were sequenced in one lane per panel on the Illumina NovaSeq PE150 platform (Illumina, San Diego, CA, USA). The resulting fastq data were subsequently uploaded to Chan Zuckerberg ID (CZID), a cloud-based metagenomics platform.

**Figure 2:**
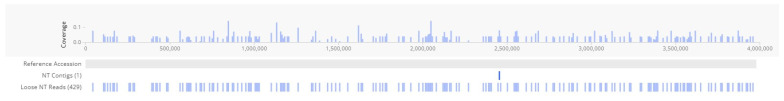
The coverage of the complete genome of *Actinomyces israelii* (GenBank: CP124548.1) (length of the reference accession: 3,980,311 base pairs) was analyzed using Chan Zuckerberg ID (CZID) Illumina mNGS pipeline v8.3.

**Figure 3:**
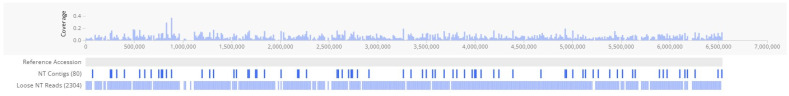
The coverage of the complete genome of *Methylobacterium* species (GenBank: CP029552.1) (length of the reference accession: 6,542,583 base pairs) was analyzed using Chan Zuckerberg ID (CZID) Illumina mNGS pipeline v8.3.

Data analysis with CZID was conducted using the Illumina mNGS pipeline v8.3 with default parameters that include human genome filtering. The analysis using a CZID pipeline gave an average percent-identity of alignments to NCBI NT/NR (%id) of 98.6% and an average expect value of alignments to NCBI NT/NR (E-value) of 10^−81^, using the complete genome of *Actinomyces israelii* (GenBank: CP124548.1) as a reference. A total of 514 reads were found to be aligned with the taxon in the NCBI NT/NR database (Figure 2), with a maximum alignment length of 263 base pairs (bp). Furthermore, the CZID pipeline gave an average percent-identity of alignments to NCBI NT/NR (%id) of 93.0% and an average expect value of alignments to NCBI NT/NR (E-value) of 10^−82^, using the complete genome of *Methylobacterium* sp. 17Sr1-1 (GenBank: CP029552.1) as a reference. A total of 21,674 reads were found to be aligned with the taxon in the NCBI NT/NR database (Figure 3), with a maximum alignment length of 918 base pairs (bp).

On 22 July 2024, based on the results of the mNGS analysis and the clinical presentation of the patient, the patient was confirmed to have cervical actinomycosis, predisposed due to the usage of steroids for De Quervain thyroiditis. Amoxicillin 2 g twice daily was started on 23 July 2024, and the wounds healed by 29 July 2024. MRI of the neck was performed at regular intervals, and the images from 31 October 2024 showed significant improvement, with complete resolution of the encasement around the right common carotid artery and the right internal jugular vein, and no irregular trans-spatial enhancing lesion noted over the right neck (Figure 1B). The patient recovered completely after 6 months of oral amoxicillin treatment. Informed consent was obtained verbally from the patient for publication of this case.

## 3. Discussion

In this report, we present a case of cervical actinomycosis diagnosed via mNGS of FFPE tissue, which is the most common clinical type of actinomycosis, accounting for approximately 50–60% of all reported cases [5,8]. The diagnosis of cervicofacial actinomycosis can be difficult. Findings in imaging are not specific, as actinomycosis is often confused with malignancy or other chronic cervicofacial infections such as nocardiosis or mycobacterial infections [8]. The gold standard for diagnosing cervical actinomycosis remains histological examination and bacterial culture. In our case, MRI of the neck confirmed the presence of an extensive irregular infiltration mass over the right side of the neck, while the histology and microbiological findings are inconclusive. In light of the lack of response to antibiotics, mNGS was chosen as a last resort to detect any potential pathogens in the specimen.

In the differential diagnosis of actinomycosis, mNGS is particularly useful in confirming the presence of *Actinomyces* species and differentiating from other similar conditions. A total of three reports published in English that utilized mNGS in the diagnosis of actinomycosis using bronchoalveolar lavage fluid (BALF) were found in PubMed (Table 1). In the case presented by Ni et al., a 64-year-old female patient was admitted with low-grade fever and chest tightness for 2 weeks [13]. The CT thorax of the patient showed diffuse ground-glass shadow in bilateral lungs [13]. Although empirical treatment with azithromycin was given, the patient did not improve clinically. Moreover, the results of the bacterial culture of the sputum and serological tests were all negative [13]. Therefore, mNGS was performed on BALF, showing the presence of the DNA of *Actinomyces odontolyticus* [13]. The patient’s condition improved after initiating penicillin G treatment and was discharged without any complications. Re-examination of the CT 3 months later revealed the resolution of lung lesions [13]. In another case presented by Kuang et al., a 66-year-old immunosuppressive male patient was admitted with cough and fever [14]. After admission, the patient produced blood-stained, grayish/yellowish sputum. The CT thorax showed diffuse ground-glass shadow in the right lung [14]. However, no pathogens were isolated from the bacterial and fungal cultures on the sputum. BALF was collected, and mNGS was performed. The diagnosis of actinomycosis was then confirmed using mNGS [14]. The clinical symptoms of the patient improved after the administration of ceftriaxone and clarithromycin [14]. Wang et al. presented a case of pulmonary actinomycosis diagnosed by a combination of radial endobronchial ultrasonography guide sheath, and mNGS in their report [15]. A 65-year-old male patient was admitted with continuous fever and dry cough [15]. The chest X-ray of the patient showed a fuzzy mass lesion in the right lung. Bronchial brushing, bronchial washing, and transbronchial biopsy were collected for microbiological investigations [15]. However, the results of conventional cultures for bacteria, fungi, and mycobacteria, and special staining were all negative. Therefore, BALF was collected for mNGS analysis, and actinomyces was detected according to the criteria of a positive mNGS result [15,16]. Thus far, clinical experience with the application of mNGS in the diagnosis of actinomycosis is still limited. All of the clinical cases mentioned in the above reports were limited to pulmonary actinomycosis. To the best of our knowledge, our case is the first case to utilize mNGS on FFPE tissue to diagnose cervical actinomycosis.

In our mNGS analysis, the DNA of two potential pathogens, namely *Actinomyces israelii* and *Methylobacterium* species, was detected. *Methylobacterium* species are strictly aerobic, fastidious, slow-growing Gram-negative bacilli [17]. *Methylobacterium* species exhibit strong biofilm-producing abilities and are major inhabitants of aqueous environments, including hospital water supply systems, medical devices, and endoscope channels [18,19,20]. The transmission of *Methylobacterium* species in a hospital environment is often associated with contaminated water systems [21]. Although the virulence of *Methylobacterium* species is low, some studies have reported healthcare-associated infections in immunocompromised patients caused by *Methylobacterium* infections [22,23,24]. In our case, considering the low virulence of *Methylobacterium* species and the absence of Gram-negative bacilli in the biopsy with Gram-staining, we believe that the detection of the DNA of *Methylobacterium* species in the mNGS analysis was due to environmental contamination by the tap water during tissue handling in the histopathology work-up. Upon review of the results of the mNGS analysis and the clinical presentations of the patient, the patient was diagnosed as having cervical actinomycosis.

In general, antibiotic resistance is not a major problem in actinomycosis, and *Actinomyces* species are usually susceptible to beta-lactam antibiotics [3], with penicillin G and amoxicillin considered the drugs of choice for the treatment of actinomycosis [3,25]. In the research performed by Moghimi et al., which included 19 cases of cervicofacial actinomycosis and 12 studies about cervicofacial actinomycosis via literature search, penicillin or amoxicillin/clavulanic acid were reported as the preferred antibiotic regimens [25]. Therefore, in our case, after confirming the diagnosis of cervical actinomycosis, oral amoxicillin treatment of amoxicillin 2 g twice daily was started, with the wound healed after a week of treatment. MRIs of the neck performed at intervals showed significant improvement after 3 months of treatment, with complete resolution of the encasement around the right common carotid artery and the right internal jugular vein, and no irregular trans-spatial enhancing lesion noted over the right neck. The patient eventually completely recovered after 6 months of oral amoxicillin treatment. This treatment outcome is comparable to those reported in the literature [25].

A novel aspect of our case is that we have shown that mNGS can be applied to FFPE tissues for pathogen detection. FFPE tissue samples are widely used in histopathology for the diagnosis of various diseases, as they allow for the long-term preservation of tissue morphology and cellular details [26]. Molecular detection of pathogens on FFPE tissue samples is challenging because the yields of microbial nucleic acids extracted from FFPE tissues are often low due to formalin fixation and dilution by a large amount of host DNA in the tissues [26,27]. Moreover, formalin fixation may result in DNA fragmentation and formalin-induced sequence artifacts [26]. Additionally, there is a risk of environmental contamination of the samples due to the nature of routine tissue handling in the histopathology work-up. Compared with PCR, which relies on the binding of primers to amplify and detect a specific segment of DNA, NGS allows for a partial analysis of the DNA fragments extracted from FFPE tissue samples to identify potential pathogens. In our case, we reported that mNGS is a promising and sensitive tool to diagnose actinomycosis. It also showed that mNGS can be applied to FFPE specimens for pathogen detection. As the first case to utilize mNGS on FFPE tissue to diagnose actinomycosis, this case report enriched the repertoire of current diagnostic methods of actinomycosis.

## 4. Conclusions

To date, actinomycosis remains a challenging condition because of its nonspecific clinical presentations and the difficulties in its accurate diagnosis. The integration of advanced diagnostic techniques such as mNGS holds promise for improving the detection and understanding of this infectious disease. As these technologies continue to evolve, they may pave the way for more effective diagnostic and therapeutic strategies and ultimately enhance patient outcomes in those affected by actinomycosis. In conclusion, this case shows that mNGS is a promising, unbiased tool for detecting *Actinomyces* species in FFPE tissues. It also highlights the diagnostic difficulties of cervical actinomycosis.

## Figures and Tables

**Table 1:** Summary of the three cases utilizing mNGS in the diagnosis of actinomycosis.

Author	Age (year)/Gender	Clinical Manifestation	*Actinomyces* Species Detected	Specimen Type
Ni et al. [13]	64/female	Low fever and chest tightness	*Actinomyces odontolyticus*	BALF
Kuang et al. [14]	66/male	High fever, cough, chest pain, and chills	Dental *Actinomyces*	BALF
Wang et al. [15]	65/male	High fever and dry cough	*Actinomyces* species *	BALF

BALF: bronchoalveolar lavage fluid. * The name ‘*Cladothrix* actinomyces’ was used in the original paper.

## Data Availability

The original contributions presented in the study are included in the article, further inquiries can be directed to the corresponding author.
